# T88. CLUSTER ANALYSIS IDENTIFIES TWO NEUROCOGNITIVE PROFILES AMONG OFFSPRING AT GENETIC RISK OF A MAJOR MENTAL DISORDER

**DOI:** 10.1093/schbul/sby016.364

**Published:** 2018-04-01

**Authors:** Rossana Kathenca Peredo Nunez de Arco, Michel Maziade, Valérie Jomphe, Elsa Gilbert, Thomas Paccalet, Chantal Merette

**Affiliations:** 1 Université Laval, CERVO Brain Research Centre; 2 CERVO Brain Research Centre; 3 Centre de Recherche CIUSSS-CN

## Abstract

**Background:**

Offspring of patients diagnosed with Schizophrenia (SZ) or Bipolar Disorder (BP) are at high risk (HR) of developing either SZ or BP and show impairment in various cognitive domains (Mortiz et al 2017, Gilbert et al 2014,). Also, the performance gradually decreases from relatives of patients with psychosis to individuals at prodromal phase and finally to subjects at first episode of psychosis (Hou et al. 2016). Recently a meta-analysis found that various cognitive domains were impaired in a pooled sample of subjects including clinical high risk for psychosis and first episode of psychosis with effect sizes ranging from -0.30 to -0.85 (Hauser et al. 2017). However, theses deficits were obtained from data of the entire sample of subjects at risk even though only a small percentage of all offspring at HR risk transit toward to a major mental disorder (Rasic et al.2014). Hence, the effect size reported may represent a mixture of larger and smaller deficits, referring to those who will eventually convert versus those who won’t, respectively. This present study addresses this issue by attempting to separate offspring of individuals with SZ or BP into two subgroups according to their cognitive profile in order to differentiate a subgroup with healthy or close to healthy cognitive performance from another having a lower performance.

**Methods:**

Our sample was composed of a HR group of 131 offspring from 6 to 24 years old. The sample was drawn from previous independent studies that targeted all multigenerational families densely affected by SZ or BP in the Eastern Québec (Canada) catchment area for genetic analysis purposes (Maziade et al. 2011). All subjects were assessed on: Processing speed, Verbal memory (VEM), Visual Memory (VISEM), Working memory and Executive functioning. An average hierarchical cluster analysis, using the Ward’s method, was performed by age group on all five cognitive domains to separate the HR group into two subgroups according to their cognitive functioning. The pseudo F statistics and Pseudo T square index were used to estimate the number of clusters and ANOVA was also performed by age group to verify that the two clusters differed in their average cognitive scores. Then, both subgroups were compared to a control group of n= 131 subjects that matched the HR group by age and gender.

**Results:**

The cluster analysis yielded two different groups, referred to as HR1 and HR2. For Processing speed and VEM, differences between HR1 and HR2 were statistically significant in almost all age groups (6-10,11-15,16–20 years old), for VISEM the two groups were different from 11 to 24 years old, while for Working memory and Executive functioning, HR1 differed from HR2 from age 16 to 24. Moreover, the HR1 group performed very similarly to the control group in all functions, while the HR2 group presented significant differences from control subjects in most cognitive performance with effect sizes often exceeding those previously seen and even reaching -2.3 for VISEM.

**Discussion:**

One of the most striking results from our study was to detect one subgroup of HR with cognitive performance very similar to non at risk individuals, while the other subgroup performed even worse than what was presented in the literature. To our knowledge, this is the first study to reveal such two neurocognitive profiles across different age groups in the HR population. Still, further research is needed in longitudinal studies to investigate whether these findings are associated with the transition to a psychiatric disorder in the following years. Nevertheless, our study suggests that interventions with a neurocognitive target should be addressed earlier, due to the apparition of a breach in cognitive performance at very early stages in life.

